# Genetic structure and diversity of natural and domesticated populations of *Citrus medica* L. in the Eastern Himalayan region of Northeast India

**DOI:** 10.1002/ece3.2174

**Published:** 2016-05-10

**Authors:** Atiqur R. Barbhuiya, Mohammed L. Khan, Selvadurai Dayanandan

**Affiliations:** ^1^ Biology DepartmentConcordia University7141 Sherbrooke Street WestMontrealQuebecH4B 1R6Canada; ^2^ Department of BotanyDr. Harisingh Gour Central UniversitySagarMPIndia

**Keywords:** Admixture, citron, diversity, domestic, Himalaya, Wild

## Abstract

Citron (*Citrus medica* L.) is a medicinally important species of citrus native to India and occurs in natural forests and home gardens in the foothills of the eastern Himalayan region of northeast India. The wild populations of citron in the region have undergone rapid decline due to natural and anthropogenic disturbances and most of the remaining individuals of citron are found in fragmented natural forests and home gardens in the region. In order to assess the genetic structure and diversity of citron in wild and domesticated populations, we analyzed 219 individuals of *C. medica* collected from four wild and eight domesticated populations using microsatellite markers. The genetic analysis based on five polymorphic microsatellite loci revealed an average of 13.40 allele per locus. The mean observed and expected heterozygosity values ranged between 0.220–0.540 and 0.438–0.733 respectively among the wild and domesticated populations. Domesticated populations showed close genetic relationships as compared to wild populations and pairwise Nei's genetic distance ranged from 0.062 to 2.091 among wild and domesticated populations. Analysis of molecular variance (AMOVA) showed higher genetic diversity among‐ than within populations. The analysis of population structure revealed five groups. Mixed ancestry of few individuals of different populations revealed exchange of genetic materials among farmers in the region. Citron populations in the region show high genetic variation. The knowledge gained through this study is invaluable for devising genetically sound strategies for conservation of citron genetic resources in the region.

## Introduction

*Citrus medica* L., commonly known as citron, is native to India (Scora 1975; Mabberley 2004) and occurs as wild and semiwild populations in both primary and secondary forests in the foothills of the Himalayas in northeast India (Hooker 1875; Bhattacharya and Dutta 1956; Tanaka 1977; Nair and Nayar 1997). Citron fruits are widely used in local medicinal practices and are a socioeconomically important genetic resource of the region. Citron is considered to have been a parental contributor to several cultivated *Citrus* accessions, and has mostly acted as the male parent (Nicolosi et al. 2000). In combination with sour orange (*Citrus *× *aurantium*), citron contributed to the origin of lemon (*Citrus limon*), bergamot (*Citrus bergamia*), and key lime (*Citrus aurantifolia*) (Barkley et al. 2006; Ollitrault et al. 2010). Natural populations of citron are severely affected by harvesting and deforestation, and most of the remaining individuals are confined to home gardens and agroforestry systems in the region. Thus, conservation measures are urgently needed to prevent further decline of citron genetic resources, and information on its genetic structure and diversity is essential for formulating conservation and management strategies.

A limited number of population genetic studies of citron using RFLP (Federici et al. 1998), RAPD, SCAR, and cpDNA (Nicolosi et al. 2000), and simple sequence repeat (SSR) and ISSR (Corazza‐Nunes et al. 2002; Barkley et al. 2006; Kumar et al. 2010; Garcia‐Lor et al. 2015) markers are reported in the literature. Through RFLP analyses, Federici et al. (1998) reported low heterozygosity levels among three *C. medica* accessions in the Citrus Variety Collection (CVC) at the University of California, Riverside. Barkley et al. (2006) studied 29 citron accessions from the CVC using SSR markers and reported lower heterozygosity values among the *C. medica* accessions as compared to the other *Citrus* species. The low genetic diversity observed among citron accessions could be attributable to selfing, as citrons are known to produce vigorous, highly homozygous seedlings through selfing (Barrett and Rhodes 1976). Genetic studies based on ISSR data also revealed a low level of heterozygosity (Ht = 0.160) in the seven accessions of *C. medica* in northeast India (Kumar et al. 2010). However, Luro et al. (2012) reported high diversity among citron varieties in the Mediterranean region, which could be attributable to intervarietal pollination and seed introductions from Asia. Using RAPD and cleaved amplified polymorphic sequence markers, Nicolosi et al. (2000) reported high genetic diversity among 12 varieties of citron. These studies are based on a limited number of *C. medica* accessions and the genetic diversity of citron in their native habitat remained unknown.

The present study, based on an extensive sampling from northeast India, is the first to assess the genetic variability of *C. medica* in its natural habitat. The overall objective of the present study is to assess the genetic diversity and structure of wild and domesticated populations of *C. medica* over a broad geographical area. The specific objectives of the present study are to (1) determine the levels of genetic diversity in wild and domesticated populations of *C. medica,* (2) determine whether the domestication process led to a reduction in genetic diversity (3) assess genetic structure and diversity of *C. medica* in its native habitat and (4) infer genetic relationships among wild and domesticated populations.

## Materials and Methods

**Figure 1:**
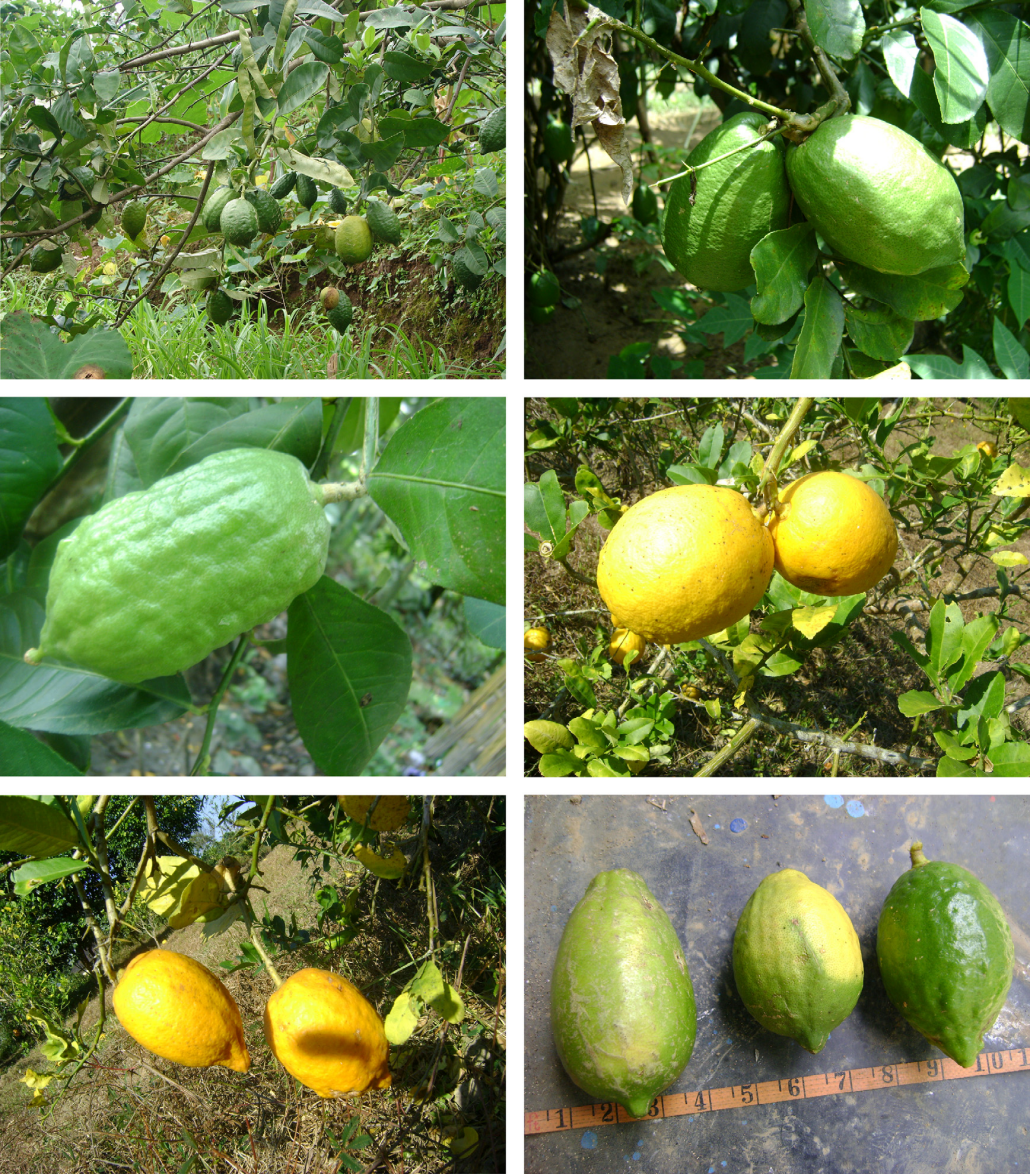
Morphological diversity of *Citrus medica* fruits in northeast India.

**Figure 2:**
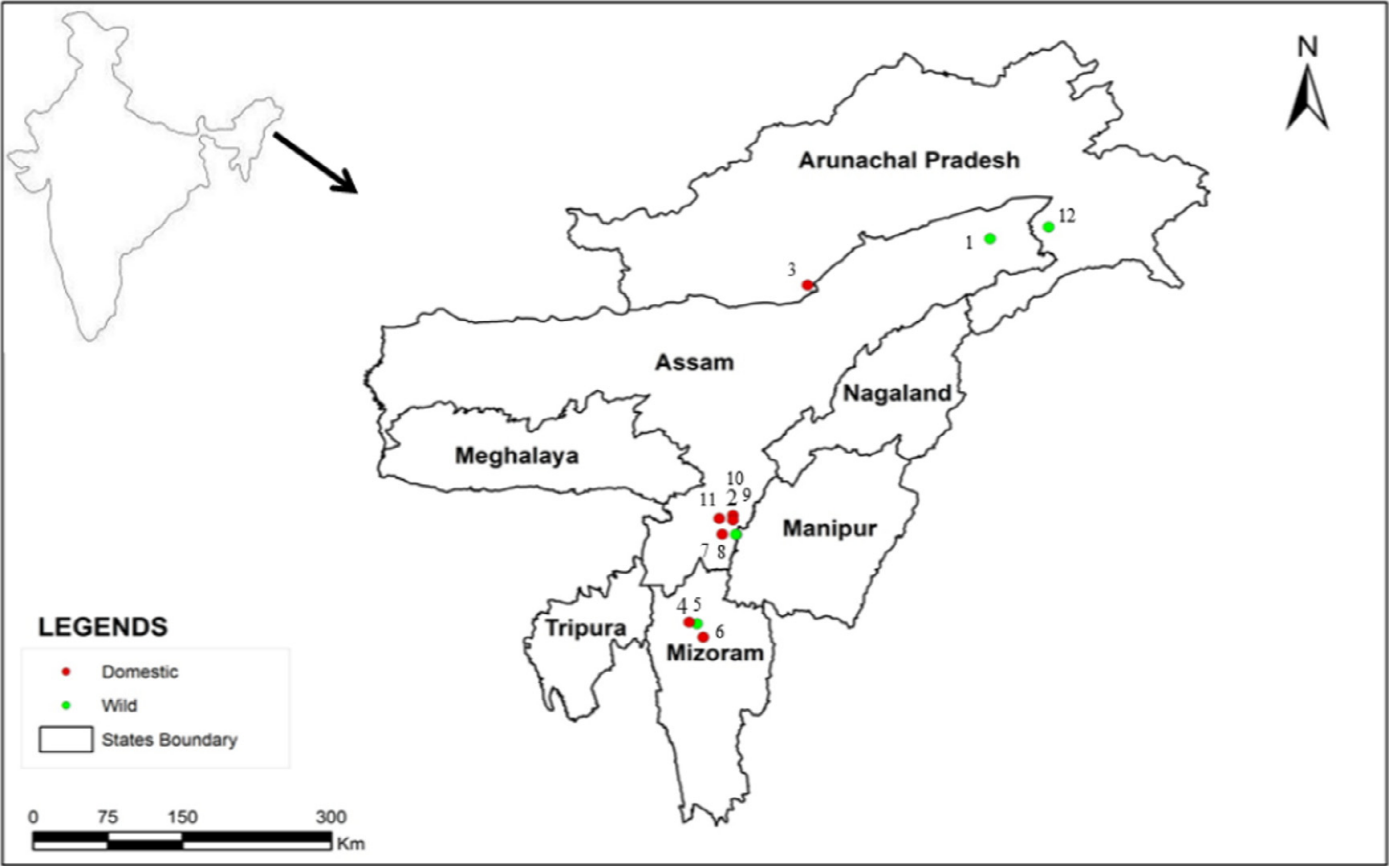
Sampling locations of *Citrus medica* populations in northeast India. Characteristics of these populations are provided in Table 1. (1) Tinsukia‐Assam, (2) Banskandi‐Assam, (3) Itanagar‐A.P., (4) Aizawl‐Mizoram, (5) Sairang^1^‐Mizoram, (6) Sairang^2^‐Mizoram, (7) Motinagar^1^‐Assam, (8) Motinagar^2^‐Assam, (9) Lakhipur‐Assam, (10) Sonai‐Assam, (11) Neairgram‐Assam, (12) Namsai‐A.P.

Leaf samples from 219 individuals of *C. medica* (Fig. 1) representing four wild and eight domesticated populations in home gardens in Assam, Arunachal Pradesh and Mizoram (Fig. 2, Table 1) were collected and stored dry until further analyses. The identification of collected samples was based on the comparison of morphological characters with those of herbarium specimens and following taxonomic monographs on *Citrus* (Bhattacharya and Dutta 1956; Tanaka 1977; Mabberley 2004). The citron members have distinct characteristics including thorny shrub to small trees; leaves are large (length 5–26 cm and width 2.5–9 cm), oblong, serrate margin, short, wingless petioles; flowers are large (3.5–6.5 cm), highly aromatic, mostly axillary racemes; fruits medium to large in size (length 2.5–12.5 cm and width 1.5–12 cm; individual fruit weight 24–210 g), shape long‐oval to ellipsoid, sometime necked, apex blunt, color green and yellow; smooth to rough fleshy thick rinds (peel thickness 0.50–3.5 cm); low juice content and highly acidic to low sweet with varied aroma, numerous seeds with white cotyledons. A total of 20 individuals per population, with the exception of Neairgram and Namsai populations where 15 and four individuals respectively were available, were sampled. Morphological features including tree height, leaf length and width, fruit shape, size and weight were recorded during sampling.

**Table 1:** Northeast India *Citrus medica* populations sampled during the present study

Population/Locality number	Source	Habitat	No. of individuals	Latitude (North) (°. ʹ. ʺ)	Longitude (East) (°. ʹ. ʺ)	Altitude (meter)
01. Tinsukia Assam	Wild	Secondary forest	20 (CM1‐20)	27.29.32.70	95.22.16.62	12
02. Banskandi Assam	Domestic	Home garden	20 (CM21‐40)	24.48.43.80	92.54.58.98	31
03. Itanagar Arunachal Pradesh	Domestic	Home garden	20 (CM41‐60)	27.06.10.41	93.41.22.32	146
04. Aizawl Mizoram	Domestic	Home garden	20 (CM61‐80)	23.43.13.45	92.42.33.46	1036
05. Sairang^1^ Mizoram	Wild	Secondary forest	20 (CM81‐100)	23.48.30.29	92.39.30.96	197
06. Sairang^2^ Mizoram	Domestic	Home garden	20 (CM101‐120)	23.48.35.19	92.39.05.12	102
07. Motinagar^1^ Assam	Domestic	Home garden	20 (CM121‐140)	24.38.38.52	92.57.51.98	35
08. Motinagar^2^ Assam	Wild	Secondary forest	20 (CM141‐160)	24.38.38.24	92.57.50.54	35
09. Lakhipur Assam	Domestic	Home garden	20 (CM161‐180)	24.47.33.74	93.00.23.13	31
10. Sonai Assam	Domestic	Home garden	20 (CM181‐200)	24.44.02.63	92.53.29.43	27
11. Neairgram Assam	Domestic	Home garden	15 (CM201‐215)	24.45.51.24	92.50.38.21	28
12. Namsai Arunachal Pradesh	Wild	Secondary forest	04 (CM216‐219)	27.40.06.48	95.51.35.13	149

Values in parentheses are the accession numbers.

The total genomic DNA from leaves was extracted following the methods of Doyle and Doyle (1987) and Dayanandan et al. (1997). The quality of extracted DNA was tested through electrophoresis on 0.5% agarose gel and staining with ethidium bromide. The PCR amplification of SSR loci was carried out following Barkley et al. (2006, 2009) and Ollitrault et al. (2010) in 15 *μ*L reactions containing 2.0 *μ*L template DNA, 0.2 *μ*L Taq polymerase, 1.5 *μ*L of 10× PCR buffer, 1.5 *μ*L of 2.5 mmol/L MgCl_2_, 1.5 *μ*L of 0.2 mmol/L dNTP, 0.5 *μ*L of the forward and reverse oligonucleotide primers (2.5 pmol each) and 0.5 *μ*L of the M13 universal forward primer (1 pmol/*μ*L), 0.5 *μ*L DMSO and 6.3 *μ*L sterile dH_2_O. Thermal cycling parameters consisted of initial denaturation at 94°C for 4 min followed by 35 cycles of 94°C for 1 min, 50–55°C for 45 sec (primer specific annealing temperature, Table 2), and 72°C for 1 min and final extension at 72°C for 7 min. PCR reactions were performed on a GeneAmp PCR System 9700 thermal cycler.

**Table 2:** Microsatellite simple sequence repeat loci used in the study

Locus	Repeat motifs	Annealing temp. (°C)	Primer sequence 5ʹ–3ʹ	Reference
cAGG9	AGG	50	F‐AATGCTGAAGATAATCCGCG	Barkley et al. (2009)
			R‐TGCCTTGCTCTCCACTCC	
CCTO1	CCT	50	F‐TCAACACCTCGAACAGAAGG	Barkley et al. (2006, 2009)
			R‐CCCACATGCTAGCACAAAGA	
GT03	GT	50	F‐GCCTTCTTGATTTACCGGAC	Barkley et al. (2006, 2009)
			R‐TGCTCCGAACTTCATCATTG	
CiBE3298	(AG)15	55	F‐TTCTCCTCCACTACACAACAC	Ollitrault et al. (2010)
			R‐CTTGAATCCCATTTCCAAC	
CiBE3936	(TC)16	55	F‐GTAATGATAGCCGTTGGTCTT	Ollitrault et al. (2010)
			R‐TATGAGATGCCTTGTATTGCT	
CiBE4796	(AG)10	55	F‐GATGAGAACGCTGATGCT	Ollitrault et al. (2010)
			R‐TTCAACCACACTGACGATAA	
CiBE0753	(AAT)13	55	F‐TCTCCTTGCCATTATTTATTT	Ollitrault et al. (2010)
			R‐CAGTTCTCAGTTGCCCGA	

Each forward oligonucleotide primer consisted of M13 tail sequence (5ʹ‐ CACGACGTTGTAAAACGAC‐3ʹ) at the 5ʹ end for visualization of the PCR product using M13 primers labeled with IRD700 and IRD800. The amplified PCR products were diluted (1:20) with loading dye (Formamide and Bromophenol blue), denatured at 94°C for 5 min and cooled on ice before loading onto the 6% polyacrylamide gel on a LI‐COR IR^2^ DNA analyzer. About 1 *μ*L aliquot of each PCR product was loaded onto each lane of the gel along with three lanes containing a 50–350 bp size standard (LI‐COR). The fragment size corresponding to each SSR marker of each sample was scored using the e‐seq software and the bands recorded as 1 (present) or 0 (absent) on an EXCEL sheet for further analysis.

### Microsatellite data analysis

The obtained genotype data for all populations and markers were tested for Hardy–Weinberg equilibrium (HWE) and linkage disequilibrium (LD) using POPGENE Version 1.31 (Yeh et al. 1999). The average number of alleles per locus (Na), the observed heterozygosity (Ho), the expected heterozygosity (He) as well as the mean number of alleles (MNA), allelic richness (*A*_R_), private allele (*A*_P_), genetic differentiation (*F*_ST_), and inbreeding coefficient (*F*_IS_) in each population and locus were calculated using software programs POPGENE Version 1.31 (Yeh et al. 1999), FSTAT version 2.9.3.2 (Goudet 2001) and Arlequin Version 3.0 (Excoffier et al. 2005). The Polymorphic Information Content (PIC) for each SSR microsatellite locus based on the entire set of accessions was calculated using Power Marker V3.25 (Liu and Muse 2005). Pairwise standard genetic distances (*D*_S_) among the 12 domesticated and wild populations were calculated following Nei's unbiased measures of genetic distance (Nei 1978) using the POPGENE software package and the resulting genetic distance matrix was used for cluster analysis through unweighted pair‐group method with arithmetic averages (UPGMA). The *F*‐statistics (*F*_IS_ = inter‐individuals, *F*_IT_ = subpopulations and *F*_ST_ = total population; Wright 1978) were computed to estimate genetic differentiation among the 12 *C. medica* populations. POPGENE Version 1.31 (Yeh et al. 1999) was used to estimate the significance of genotypic differentiation between population pairs. All probability tests were based on the Markov chain method (Guo and Thompson 1992; Raymond and Rousset 1995) using 1000 dememorization steps, 100 batches and 1000 iterations per batch. When the null hypothesis was rejected, the *F*_IS_ statistic of Wright (1951) was estimated following Weir and Cockerham (1984) and used as an indicator of heterozygote excess or deficit. The *F*_ST_ statistic (Wright 1951) was estimated following Weir and Cockerham (1984) and pairwise tests of differentiation were performed in FSTAT. Permutation tests were performed in FSTAT, where genotypes were randomized among samples and the significance of the *P*‐values from the pairwise tests of differentiation was determined using standard Bonferroni corrections.

Analysis of molecular variance (AMOVA) (Excoffier et al. 1992) was performed in Arlequin 3.0 software (Excoffier et al. 2005) to test the differentiation of the accessions in various groups with the probability of nondifferentiation (*F*_ST_ = not > 0) over 10000 randomizations. The distribution of genetic variation within and among wild and domesticated populations was estimated using Nei's standard genetic variation (Nei 1987). Pairwise *F*_ST_ values between all pairs of populations were calculated and differentiations were tested between the populations in Arlequin. To examine the geographic structure of genetic variation among the *C. medica* populations, we tested for correlations between genetic distance and geographic distance using a Mantel test based on a pairwise matrix of Nei's (1978) unbiased genetic distances, Rousset (1997) genetic differentiation [*F*_ST_/(1 − *F*_ST_)] and a pairwise matrix of geographic distances (Mantel 1967). Gene flow (Nm) among populations was estimated as the number of migrants per generation between pairs of populations. Nm was estimated according to Slatkin (1993) by using the formula Nm = (1−F_ST_) /4F_ST_.

Genetic bottlenecks among populations were identified using the program BOTTLENECK version 1.2.02, under three different models, the infinite allele and stepwise mutation (Cornuet and Luikart 1996), and the two‐phased model of mutation (Luikart et al. 1998). Both the Wilcoxon signed‐rank test and a sign test were used to assess significance of whether the observed He is greater than expected under an equilibrium model.

The software program STRUCTURE version 2.1 (Pritchard et al. 2000) was used for the analysis of population structure and identification of ancestral and hybrid forms. This method follows a Bayesian clustering approach to assign individuals into clusters using multilocus genotype data and allele frequencies. This approach works on the principle that the loci selected for investigation are unlinked, independent and at linkage equilibrium among the populations under the Hardy–Weinberg principle (Pritchard et al. 2000). Different accessions were assigned to probable clusters under the assumption that all accessions were from a common ancestor and that admixing of individuals among the populations had occurred. The posterior probabilities were estimated using a Markov Chain Monte Carlo (MCMC) method. The admixture of individuals independent of the geographic locations was used for clustering all individuals from the study populations and 15 independent runs of STRUCTURE were carried out for the total data set for *K* (number of clusters) values of 1–15. Simulations were carried out with the following settings: admixture model, correlated allele frequencies, and MCMC repetitions of 10,000 iterations. The final results were based on a run length of 100,000 and five iterations for each *K* using admixture model with the independent frequency and correlation model. We examined *ΔK* values, which are derived from the second‐order rate of change of the likelihood function used to determine *K* (Evanno et al. 2005), to provide a better estimate of the number of clusters in such conditions. For the number of clusters best represented by the data, only individuals with probabilities above the threshold *q* = 0.75 for a specific cluster were retained in that population.

## Results

Characteristics of the seven SSR markers used to assess genetic diversity of the 219 *Citrus medica* individuals are given in Table 2. Five of the seven primer pairs described by Barkley et al. (2006, 2009) and Ollitrault et al. (2010) were used for genetic analysis. Two of the seven markers, cAGG9 and CCTO1, were excluded from the analysis due to their low polymorphism and poor amplifications. All SSR loci used in the present study were polymorphic and none of the loci deviated from Hardy–Weinberg equilibrium. No significant LD was found in any pairs of loci, so all five SSR microsatellite loci provided independent information. A total of 67 alleles were detected within the citron individuals, with allele frequencies across all loci ranging from 2.50% to 82.50%. The number of alleles generated by each SSR marker varied from eight to 20 with an average of 13.4 alleles per locus (Table 3). The highest number of alleles was scored at locus CiBE3936 (20 alleles) and lowest number of alleles at locus CiBE4796 (8 alleles) (Table 3). The effective number of alleles (Ne) for each locus ranged from 3.66 to 6.25 with an average value of 4.85. The amplified fragment size of the alleles varied from 131 (CiBE3936) to 248 (CiBE3298) bp. The PIC values ranged between 0.829 (CiBE3936) and 0.694 (CiBE0753) with a mean PIC value of 0.762 for all loci (Table 3).

**Table 3:** Diversity statistics of the five polymorphic simple sequence repeat loci used among 219 *Citrus medica* individuals. Statistics include number of alleles (Na), polymorphic information content (PIC), effective number of alleles (Ne), observed (Ho) and expected (He) heterozygosity, Nei's standard genetic distance (*D*_S_), local inbreeding coefficient (*F*_IS_), overall inbreeding coefficient (*F*_IT_), genetic differentiation (*F*_ST_) and gene flow (Nm)

Locus	Na	PIC	Ne	Ho	He	*D*_S_	*F*_IS_	*F*_IT_	*F*_ST_	Nm
GT03	12	0.773	4.91	0.369	0.798	0.796	0.373	0.556	0.292	0.606
CiBE3298	9	0.752	4.67	0.438	0.788	0.786	0.281	0.438	0.219	0.891
CiBE3936	20	0.829	6.25	0.532	0.842	0.84	0.266	0.375	0.149	1.426
CiBE4796	8	0.761	4.78	0.379	0.793	0.791	0.204	0.461	0.323	0.522
CiBE0753	18	0.694	3.66	0.196	0.728	0.727	0.548	0.725	0.391	0.387
Mean		0.762	4.85	0.383	0.79	0.788	0.334	0.511	0.275	0.767
±SD		0.048	5.36	0.123	0.041	0.04	0.133	0.136	0.093	0.184

The total number of alleles across all loci ranged between 13 in the Namsai wild population and 36 in the Banskandi domesticated population. The mean allelic richness (*A*_R_), independent of sample size, ranged between 3.83 in the Tinsukia wild population to 2.48 in the Sairang^2^ domesticated population (Table 4). Overall, genetic diversity varied significantly within wild and domesticated populations located in different geographic locations. The MNA across all populations was 2.77 ± 0.17, varying between 2.60 ± 0.55 in the Namsai wild population, which had the lowest number of individuals (4), and 7.20 ± 2.95 in the domesticated Banskandi population. In general, a higher MNA was observed in the domesticated populations. Most of the alleles present in domesticated populations were also present in wild populations. Private alleles, unique to a specific population, were observed in the Itanagar domesticated population (*A*_P_ = 4), as well as in the Tinsukia wild, Banskandi domesticated, Aizawl domesticated and Sairang^1^ wild populations, each with two private alleles, and in the Sairang^2^ and Motinagar^1^ domesticated populations, which had one private allele each. No private alleles were found in any of the other populations (Table 4). The frequencies of these private alleles ranged between 2.50–12.50%.

**Table 4:** Diversity statistics by *Citrus medica* population. Statistics include allelic richness (*A*_R_), number of private alleles (*A*_P_), mean number of alleles (MNA), polymorphic information content (PIC), observed (Ho) and expected (He) heterozygosity, genetic differentiation (*F*_ST_ = average of pairwise *F*_ST_), local inbreeding coefficient (*F*_IS_ = 1 − Ho/He) and gene flow (Nm = (1 − *F*_ST_)/4*F*_ST_)

Population	*A*_R_	*A*_P_	MNA	PIC	Ho	He	*F*_ST_	*F*_IS_	Nm
01	3.83 ± 0.99	2	5.60 ± 2.30	0.672	0.220 ± 0.160	0.733 ± 0.093	0.174 ± 0.092	0.705***	1.187
02	3.76 ± 1.08	2	7.20 ± 2.95	0.640	0.540 ± 0.251	0.706 ± 0.107	0.193 ± 0.099	0.239***	1.045
03	3.43 ± 1.16	4	6.00 ± 3.08	0.583	0.390 ± 0.249	0.658 ± 0.127	0.199 ± 0.117	0.413**	1.006
04	3.19 ± 1.01	2	6.00 ± 2.35	0.538	0.470 ± 0.299	0.603 ± 0.202	0.217 ± 0.119	0.224***	0.902
05	3.06 ± 0.65	2	5.80 ± 2.05	0.505	0.400 ± 0.158	0.555 ± 0.158	0.236 ± 0.132	0.285***	0.809
06	2.48 ± 0.68	1	4.00 ± 1.41	0.389	0.330 ± 0.279	0.438 ± 0.217	0.294 ± 0.177	0.252***	0.600
07	3.26 ± 1.15	3	5.40 ± 2.61	0.539	0.269 ± 0.213	0.600 ± 0.165	0.218 ± 0.128	0.559***	0.902
08	3.11 ± 0.51	–	4.40 ± 0.55	0.552	0.372 ± 0.333	0.622 ± 0.117	0.252 ± 0.106	0.399**	0.742
09	2.81 ± 0.85	–	4.80 ± 1.92	0.458	0.410 ± 0.185	0.512 ± 0.188	0.268 ± 0.132	0.204**	0.683
10	3.01 ± 0.98	–	4.40 ± 1.52	0.515	0.360 ± 0.225	0.580 ± 0.223	0.214 ± 0.126	0.385**	0.918
11	2.83 ± 0.70	–	3.80 ± 1.48	0.507	0.453 ± 0.321	0.604 ± 0.094	0.258 ± 0.129	0.256**	0.719
12	2.60 ± 0.54	–	2.60 ± 0.55	0.375	0.450 ± 0.326	0.500 ± 0.152	0.249 ± 0.146	0.115^ns^	0.754

±: standard deviation.

(1)Tinsukia‐Assam, (2) Banskandi‐Assam, (3) Itanagar‐A.P., (4) Aizawl‐Mizoram, (5) Sairang^1^‐Mizoram, (6) Sairang^2^‐Mizoram, (7) Motinagar^1^‐Assam, (8) Motinagar^2^‐Assam, (9) Lakhipur‐Assam, (10) Sonai‐Assam, (11) Neairgram‐Assam, (12) Namsai‐A.P.

Significance levels: **P < 0.01, ***P < 0.001; ns: non‐significant.

The mean observed (Ho) and expected (He) heterozygosity values varied significantly (*P* < 0.001) within the populations (Table 4). The highest value for Ho = 0.540 ± 0.251 was observed in the domesticated Banskandi population, while the lowest Ho = 0.220 ± 0.160 occurred in the Tinsukia wild population. The highest He within the populations was found in the Tinsukia wild population (He = 0.733 ± 0.093), while the lowest occurred in the Sairang^2^ domesticated population (He = 0.438 ± 0.217). The He values for wild populations ranged from 0.500–0.733, and for domesticated populations it ranged from 0.438–0.706. This wide range of heterozygosity values indicates high diversity within the populations. In all cases, average observed heterozygosities were lower than the expected heterozygosities under HWE (Table 4).

Population differentiation (*F*_ST_) values were calculated for each locus and population separately and slight variation was observed among loci (Table 3) and populations (Table 4). The *F*_ST_ values ranged between 0.174–0.252 in wild populations and 0.193–0.294 in domesticated populations, with slightly greater values in domesticated populations. The *F*_ST_ values and their level of significance for pairs of populations were also calculated (Table 5). Among the 12 pairs of populations, only three pairs were not significantly differentiated, viz., Banskandi (domesticated) and Tinsukia (wild), Aizawl and Itanagar (domesticated) and Sairang^1^ (wild) and Sairang^2^ (domesticated). All other population pairs were significantly differentiated and the significance level in the most of the population pairs was *P* < 0.001 (Table 5). The greater and significant *F*_ST_ values between these population pairs may indicate greater genetic divergence in citron populations among these pairs. Inbreeding coefficient (*F*_IS_) values were significantly positive (*F*_IS_ = 0.204–0.705; 0.001 < *P* < 0.05) for all the populations except for one wild population in which it was positive but insignificant (*F*_IS_ = 0.115; *P* > 0.05) (Table 4). In all loci, significantly positive *F*_IS_ values were obtained and these ranged between 0.204–0.548. The average value of *F*_IS_ for all loci was 0.334 and *F*_IT_ was 0.511 for all accessions (Table 3). The gene flow (Nm) was calculated according to genetic differentiation and it ranged between 0.600 in the Sairang^2^ domesticated population to 1.187 in the Tinsukia wild population (Table 4).

**Table 5:** Pairwise genetic differentiation (*F*_ST_) (below the diagonal) and Nei's standard genetic distance (*D*_S_) (above the diagonal) among the 12 *Citrus medica* populations

	1	2	3	4	5	6	7	8	9	10	11	12
1	–	0.202	0.355	0.391	0.797	0.801	0.689	1.597	1.174	0.641	0.558	0.577
2	0.048^ns^	–	0.324	0.349	0.886	0.828	0.712	1.375	1.007	0.806	1.138	0.841
3	0.103**	0.101**	–	0.078	0.600	0.527	0.467	1.559	0.962	0.968	1.752	1.331
4	0.130**	0.124**	0.022^ns^	–	0.774	0.703	0.579	1.475	0.773	0.717	1.487	1.013
5	0.230***	0.253***	0.219***	0.276***	–	0.062	0.146	1.011	1.255	1.147	1.386	1.262
6	0.285***	0.299***	0.252***	0.315***	0.041^ns^	–	0.079	1.387	1.768	1.792	2.091	1.952
7	0.192**	0.206***	0.169**	0.217***	0.067*	0.058*	–	1.174	1.236	1.297	1.893	1.640
8	0.273***	0.271***	0.305***	0.325***	0.305***	0.400***	0.297***	–	0.207	0.483	0.964	0.751
9	0.295***	0.288***	0.302***	0.295***	0.382***	0.477***	0.355***	0.112**	–	0.185	0.953	0.570
10	0.194**	0.231***	0.272***	0.255***	0.339***	0.445***	0.328***	0.194**	0.108*	–	0.337	0.156
11	0.165**	0.257***	0.322***	0.335***	0.351***	0.452***	0.355***	0.276***	0.326***	0.151**	–	0.249
12	0.174**	0.234***	0.317***	0.314***	0.373***	0.503***	0.375***	0.263***	0.275***	0.055*	0.101*	–

(1) Tinsukia‐Assam, (2) Banskandi‐Assam, (3) Itanagar‐A.P., (4) Aizawl‐Mizoram, (5) Sairang^1^‐Mizoram, (6) Sairang^2^‐Mizoram, (7) Motinagar^1^‐Assam, (8) Motinagar^2^‐Assam, (9) Lakhipur‐Assam, (10) Sonai‐Assam, (11) Neairgram‐Assam, (12) Namsai‐A.P.

Significance levels: *P < 0.05; **P < 0.01, ***P < 0.001; ns: non‐significant.

The pairwise Nei's genetic distance (*D*_S_) values are summarized in Table 5. In general, domesticated populations showed close genetic relatedness as compared to wild populations. The pairwise *D*_S_ vales between populations ranged from 0.062 between the Sairang^1^ wild and Sairang^2^ domesticated populations in Mizoram to 2.091 between two domesticated populations, Sairang^2^ (Mizoram) and Neairgram (Assam). Similar results were observed when the genetic distances of the populations in the study were determined using Nei's *D*_A_ index (Nei's unbiased genetic distance) of genetic distance. The smallest *D*_A_ was observed between the Sairang^1^ wild and Sairang^2^ domesticated populations (0.049) and largest *D*_A_ was observed between the Neairgram and Sairang^2^ domesticated populations (2.074) (Data not shown here). The AMOVA showed significant total genetic variation among the populations and individuals (*P* < 0.001) for all variance components. The genetic differences were 27.49% among individuals within populations, 24.98% among populations, and 47.53% at the individual level (Table 6).

**Table 6:** Summary of analysis of molecular variance (AMOVA) for 12 populations and 219 individuals

Source of variation	DF	Sum of squares	Variance components	Percentage of variation (%)	Fixation indices	*P* value
Among populations	11	222.886	0.501	24.98	*F*_ST_ = 0.249	0.001
Among individuals within populations	207	426.092	0.552	27.49	*F*_IS_ = 0.366	0.001
Within individuals	219	209.00	0.950	47.53	*F*_IT_ = 0.524	0.001

**Figure 3:**
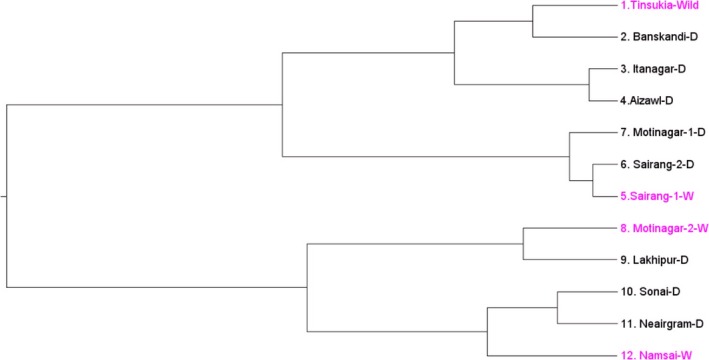
Unweighted pair‐group method with arithmetic averages dendrogram representing genetic relationships among the 12 *Citrus medica* populations, constructed using Nei's genetic distance calculated from allele frequencies observed at five microsatellite loci

Genetic relatedness between wild and domesticated populations was determined using Nei's standard and unbiased genetic distances and UPGMA methods. The UPGMA dendrogram showed five different clusters of *C. medica* accessions for all 12 populations and there was an admixture of individuals between wild and domesticated populations. The first cluster comprised two geographically isolated populations, Tinsukia (wild) and Banskandi (domesticated); the second cluster consisted of distant populations Itanagar and Aizawl (both domesticated); the third cluster contained the Sairang^2^ (wild), Sairang^1^ (domesticated), and Motinagar^1^ (domesticated) populations, which are located in the same geographic region; the fourth cluster was formed by the Motinagar^2^ (wild) and Lakhipur (domesticated) populations; and the fifth cluster was made up of two proximate domesticated populations Sonai and Neairgram and the distant, wild Namsai population (Fig. 3).

**Figure 4:**
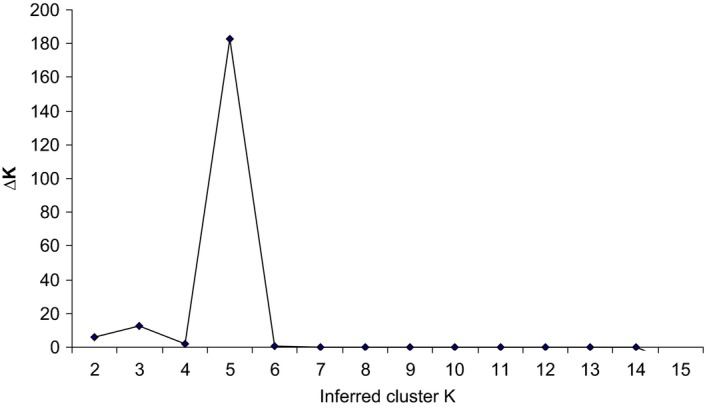
The number of inferred clusters *K* based on mean log likelihood probability values (∆*K*) (*K* = 1–15) obtained from STRUCTURE analysis. The most likely value for putative population identified at *K* = 5.

**Figure 5:**
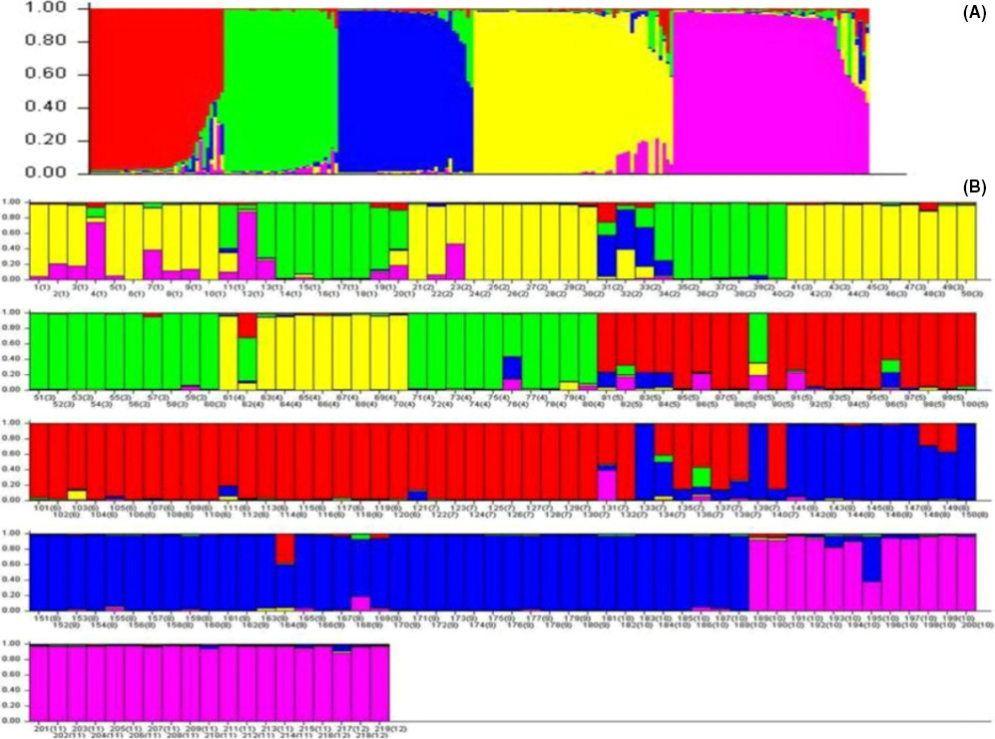
Population assignments by STRUCTURE. (A) Clustering of populations at *K* = 5. The *X*‐axis shows population numbers as defined in Table 1; the *Y*‐axis shows the proportion of alleles derived from each population. Accession assignments are as follows (population numbers and proportion): Cluster 1: #5 (34%), #6 (36%) and #7 (30%) Cluster 2: #1 (24%), #2 & #3 (26% each) and #4 (24%); Cluster 3: #2 & #7 (6% each), #8 & #9 (36% each) and #10 (16%). Cluster 4: #1 (24%), #2 (18%), #3 (26%), #4 (29%) and #5 (3%); and Cluster 5: #1 (4%), #10 (19%), and #11 & #12 (38.5% each) (B) Assignment of 219 individual (population number in brackets) *Citrus medica* accessions to into five distinct clusters. The *Y*‐axis shows the proportion of alleles derived from each individual. Individuals of the same color belong to the same cluster. An individual with more than one color shares a percentage of its among multiple clusters, according to the admixture proportions.

The STRUCTURE analysis revealed five distinct clusters (*K* = 5) represented by the individuals having posterior probability values above the threshold value *q* = 0.75 (Fig. 4). The assignment of individuals into wild and domesticated population groups is presented in Figure 5. Bayesian clustering analysis assigned 219 accessions into five genetically inferred clusters. Cluster 1 mainly comprises individuals of three different populations, among them one wild population, #1 (34%), and two geographically isolated domesticated populations, #6 (36%) and, #7 (30%). Cluster 2 is dominated by individuals of three geographically isolated domesticated populations #2 and #3 (26% each) and #4 (24%), and one distant wild population, #1 (24%). Cluster 3 contains individuals belonging to same geographic location of four domesticated populations # 2 and # 7 (6% each), # 9 (36%), and one wild population # 8 (36%). Cluster 4 has individuals from one distant wild population, #1 (24%), and three distantly located domesticated populations, #2 (18%), #3 (26%) and #4 (29%). In cluster 5, the majority of the accessions were contributed by two geographically isolated domesticated and wild populations #11 (38.5%) and #12 (38.5%) and two other populations # 1 (4%) and #10 (19%). (Table 7; Fig. 5).

**Table 7:** Proportion of ancestry of each population in each of the gene pools as defined using the model‐based clustering method from Pritchard et al. (2000)

Populations/Clusters	Proportion of individuals in each gene pool (%)											
	P1	P2	P3	P4	P5	P6	P7	P8	P9	P10	P11	P12
Cluster 1	–	–	–	–	34	36	30	–	–	–	–	–
Cluster 2	24	26	26	24	–	–	–	–	–	–	–	–
Cluster 3	–	6	–	–	–	–	6	36	36	16	–	–
Cluster 4	24	18	26	29	3	–	–	–	–	–	–	–
Cluster 5	4	–	–	–	–	–	–	–	–	19	38.5	38.5

(1) Tinsukia‐Assam, (2) Banskandi‐Assam, (3) Itanagar‐A.P., (4) Aizawl‐Mizoram, (5) Sairang^1^‐Mizoram, (6) Sairang^2^‐Mizoram, (7) Motinagar^1^‐Assam, (8) Motinagar^2^‐Assam, (9) Lakhipur‐Assam, (10) Sonai‐Assam, (11) Neairgram‐Assam, (12) Namsai‐A.P.

Correlation between geographic distance (km) and Nei's genetic distance among the citron populations of NE India was insignificant. The geographic distance among the populations ranges from 0.01 to 535 km. Mantel test also showed no significant correlation between geographic distance and genetic differentiation [*F*_ST_/(1 − *F*_ST_)] for *C. medica* populations in the region. Thus, genetic distances between populations are independent of the corresponding geographical distances.

## Discussion

The present study is the first to quantify the amount and distribution of genetic variability in *C. medica* within its native geographical range. The results, based on genotypes of five selected SSR loci, demonstrate that domesticated citron populations possess a slightly higher genetic diversity than wild populations and the difference between those populations was insignificant. High levels of polymorphism in the five selected SSR markers allowed us to unambiguously distinguish 219 accessions belonging to 12 geographically isolated populations.

Overall diversity values obtained in the present study differ from those found by Ollitrault et al. (2010), who reported low genetic diversity (He = 0.15, 1.44 alleles per locus). A prior study by Barkley et al. (2006) also reported lower diversity indices between citron individuals. In a recent microsatellite marker based study of 47 citrons from Yunnan Province of China and Mediterranean region by Ramadugu et al. (2015) and 56 citron from southwest China by Yang et al. (2015) reported substantial heterozygosity and genetic diversity among citron accessions. These differences in genetic diversity between the present and previous studies could be attributable to sample size as limited number of individuals were sampled in earlier studies. More importantly, current sampling from different regions throughout its native range, rather than from small numbers of accessions in ex situ germplasm banks may have resulted in a better assessment of the genetic diversity present in *C. medica*. These results show that there are abundant genetic variation at the molecular level among the 219 citron individuals from four wild and eight domesticated populations throughout northeast India, where the species are thought to have originated. A large number of studies suggest that the primary centre of origin of *Citrus* is south and south‐east Asia, particularly the region extending from northeast India, eastward through the Malayan Archipelago to China and Japan, and southward to Australia (Tanaka 1958; Swingle and Reece 1967; Scora 1975; Mabberley 2004). Extensive field exploration studies and presence of large number of natural populations in the primary forests also revealed that the region is the center of origin of several *Citrus* species (Bhattacharya and Dutta 1956).

The domesticated populations of *C. medica* have slightly higher genetic diversity as compared to those wild populations. In general, all the populations have lower observed heterozygosity values then the expected heterozygosity suggesting inbreeding. Slightly higher genetic diversity among the domesticated populations suggest that movement of cultivated individuals through a large geographic distances resulting in allele combinations which would not occur naturally (Miller and Gross 2011). The exchange of such highly valued medicinal plants in the form of seed, seedlings and mature plant cuttings, sometimes over long distances, is a common practice among tribal and nontribal communities in the region. Most likely farmers may have selected individuals with desirable traits, which may have contributed to the increased genetic diversity in domesticated populations through increased mixing and gene flow among geographically isolated populations.

An average *F*_ST_ = 0.275 for overall loci revealed significant genetic differentiation between populations. Similar moderate‐to‐high *F*_ST_ values are consistent with the relatively high genetic differentiation observed in some other tropical trees *Caryocar brasiliense* (Collevatti et al. 2001), *Swietenia macrophylla* (Novick et al. 2003), and *Dalbergia monticola* (Andrianoelina et al. 2009). These results also reflect genetically distinct populations in the region differing simultaneously in allele frequencies and allele sizes, and suggest that new mutations may be contributing to the allelic diversity found in wild and domesticated citron populations. In general, wild and domesticated citron populations showed strong genetic differentiation. Domesticated populations showed a higher proportion of genetic differentiation (*F*_ST_ = 0.193–0.294) than wild populations (*F*_ST_ = 0.174–0.252). Similarly, Hamrick and Godt (1996) reported that the mean value of genetic differentiation among populations of crop species (domesticated) is higher than that of noncrop (wild) species. The observed high *F*_ST_ values in cultivated populations can be explained by distinct sources of germplasm used in establishing domesticated populations with limited exchange of genetic material, leading to high genetic differences among domesticated populations. The results are supported by the long cultivation history of citron species in the region. Some of the domesticated populations are not far from wild habitats; therefore, migration from wild to cultivated populations by natural or artificial means may be an ongoing process. Abundant occurrences of wild and primitive relatives of citron, e.g., *C. nana* (Wester) Yu.Tanaka, *C. odorata* (Wester) Tanaka and species under the subgenus *Papeda* in the eastern Himalayan areas (Tanaka 1969), as well as our recent *Citrus* germplasm collection in northeast India indicate their persistence and diversification in the region of origin. Favorable environmental conditions in this area, currently in the ‘Indo‐Burma biodiversity hot spot’ favored its growth and further spreading to other parts of the world (Tanaka 1969). In a recent palynological study, Langgut (2014) stated that citron originated in Asia, particularly India and then gradually dispersed to other areas.

The AMOVA results revealed a high level of genetic variation among individuals (47.53% of the total variation) and significantly (*P* < 0.001) low level of variation among populations (24.98%). In most of the citron populations, seeds or cuttings of one or a few individuals were brought from the wild population, transferred to and grown in the farmers' home gardens or local agroforestry systems, and maintained for generation after generation. In clonally propagated plants, separation from the wild ancestor during the domestication process reduces the chances of sexual crossing in subsequent populations (Zohary and Spiegel‐Roy 1975; McKey et al. 2010). However, in many perennial plant species heterozygosity also maintained through clonal propagation (Petit and Hampe 2006). Clonal propagation methods may have increased the homogeneity at the population level. The citron populations showed significant inbreeding coefficients (*F*_IS_) (*P* < 0.001–0.01), with the single exception of the Namsai wild population.

The indirect estimates of geneflow (Nm) based on population differentiation showed significant variation (*P* < 0.001) and ranged between 0.600 and 1.187. Population differentiation and effective population size corresponded to three different categories of Nm values: high (Nm ≥ 1.000), intermediate (0.250–0.990) and low (0.000–0.249) (Slatkin 1981, 1985). One wild population, Tinsukia, and three domesticated populations, Banskandi, Itanagar and Aizawl, showed relatively high gene flow (Nm > 1.000) and in the other populations it was intermediate (Nm = 0.600–0.918). The relatively high through intermediate levels of gene flow among populations attributable to the movement of genetic material among farmers in the region. Genetic distances between wild and domesticated populations are smaller and admixture is more common between sympatric populations of wild and domesticated populations than between allopatric populations, which is indicative of gene flow between sympatric populations. The presence of a few private alleles (1–4) in most of the wild and domesticated populations also shows the existence of gene flow among populations (Slatkin 1985). A review by Ellstrand et al. (1999) of thirteen globally important crops including wheat, rice and maize concluded that gene flow among wild and domesticated relatives is common and unintentional, and occurs naturally whenever these relatives come into contact with each other. Viard et al. (2004) and Scurrah et al. (2008) reported similar results of gene flow among the wild and domesticated annual crop plants (beet and potato species) through seeds and clonal propagation. Similar results have also been reported for many perennial food plants (Miller and Gross 2011).

The BOTTLENECK analysis indicated that no bottleneck event occurred in citron populations of the region. It is possible that slight or past bottleneck effects may have gone undetected. A number of natural citron populations in the region have diminished, due to natural and anthropogenic disturbances and overexploitation. Until now, such disturbances have had no identifiable consequences in terms of overall genetic diversity and effective population size. Citron populations in the region are maintaining their allelic richness without any reduction in genetic diversity through either natural processes or farming methods. Future studies on larger populations and a wider selection of markers and methods are needed to detect bottleneck events.

The STRUCTURE analysis showed shared ancestry between the wild and domesticated citron populations, suggesting that gene flow has occurred between these populations. Overall, the STRUCTURE results suggest five subpopulations within the 12 wild and domesticated populations. The grouping of individuals into five distinct clusters is supported by the highest *∆K* value, confirming the presence of five genetically distinct groups (Fig. 4 and Table 7). This is further supported by AMOVA, which showed that most of the total variance distributed within individuals (47.53%) and among individuals within populations (27.49%). A few individuals of some populations genetically related to individuals of geographically isolated populations of the region. Similar groupings through cluster analysis also supports gene flow among distant populations. The genetic diversity observed among the wild and domesticated populations did not affect the clustering of the individuals at the population level. Grouping of wild and domesticated individuals in to the same cluster indicates their admixture due to the long history of cultivation in the region. The domesticated Banskandi population and the wild Tinsukia population showed similarly large amounts of genetic diversity; however, most of the individuals from these two distant populations clustered together (Cluster‐1 and 3, Fig. 5). Such clustering suggest admixture of individuals among distant populations, which could be attributable to the long history of genetic material exchange. Individuals of *C. medica* may have spread from wild sources, (i.e., the site of origin), to farmer‐managed lands through the movement of the people or sharing of seeds. Further, the UPGMA dendrogram (Fig. 3) clustered 12 populations into five groups. The cluster analysis could not clearly differentiate the wild and domesticated populations. Thus, there has been mixing of wild and domesticated populations. The nonsignificant (*P* > 0.05) relationship between geographic and genetic distances between populations indicates that their genetic differences are independent of corresponding geographical distances.

## Conclusion

There is a significant level of genetic diversity in the citron germplasm that could be used for sustainable utilization and conservation of this valuable genetic resource. The Himalayan northeast region of India is believed to be a center of diversity for the genus *Citrus* and this study reveals that high level of genetic diversity exists in *Citrus medica*. This also supports the views of Vavilov (1951) who stated that generally plant species show high diversity in their original place of origin and in the regions with large number of wild relatives of crop plants. A few individuals showed mixed ancestry between the wild and domesticated populations. The observed intraspecific genetic variation in the citron germplasm is valuable for selecting the most diverse populations for further improvement of fruit quality through breeding programmes and commercialization. The present study shows that the genetic diversity of *Citrus medica* has been maintained by the indigenous communities in their home gardens through traditional cultivation practices. This highlights the important role played by indigenous communities in conservation of genetic resources.

## Conflict of Interests

The authors declare that they have no competing interests.

## Supporting information


**Table S1**. *Citrus medica* population sampling details (Geographical location information is in Table 1).
**Table S2.** Genotypes of *Citrus medica* population in NE India.
**Table S3.** Allele frequency comparison over populations.
**Figure S1.** Relationship between geographic distance and Nei's genetic distance among the 12 populations of wild and domestic *C. medica*.
**Figure S2.** Relationship between geographic distance and genetic differentiation [*F*_ST_/(1 − *F*_ST_)] among the 12 populations of wild and domestic *C. medica*. *F*_ST_ was calculated according to Weir and Cockerham (1984).
