# Supplementary material for: Genetic structure and diversity of natural and domesticated populations of Citrus medica L. in the Eastern Himalayan region of Northeast India
Source: Ecol Evol. 2016 May 10;6(12):3898–911. doi: 10.1002/ece3.2174 (PMC4972219; doi:10.1002/ece3.2174)
Supplement: Supplementary file 1 — Table S1. Citrus medica population sampling details (Geographical location information is in Table 1). Table S2. Genotypes of Citrus medica population in NE India. Table S3. Allele frequency comparison over populations. Figure S1. Relationship between geographic distance and Nei's genetic distance among the 12 populations of wild and domestic C. medica. Figure S2. Relationship between geographic distance and genetic differentiation [F ST/(1 − F ST)] among the 12 populations of wild and domestic C. medica. F ST was calculated according to Weir and Cockerham (1984). [file ECE3-6-3898-s001.docx]

**Supplementary Table 1**.*Citrus medica* population sampling details (Geographic information are there in Table 1)

Accessions Location: Source or Habitat Accessions CODE

C. medica Tinsukia-Assam Wild CM1

C. medica Tinsukia-Assam Wild CM2

C. medica Tinsukia-Assam Wild CM3

C. medica Tinsukia-Assam Wild CM4

C. medica Tinsukia-Assam Wild CM5

C. medica Tinsukia-Assam Wild CM6

C. medica Tinsukia-Assam Wild CM7

C. medica Tinsukia-Assam Wild CM8

C. medica Tinsukia-Assam Wild CM9

C. medica Tinsukia-Assam Wild CM10

C. medica Tinsukia-Assam Wild CM11

C. medica Tinsukia-Assam Wild CM12

C. medica Tinsukia-Assam Wild CM13

C. medica Tinsukia-Assam Wild CM14

C. medica Tinsukia-Assam Wild CM15

C. medica Tinsukia-Assam Wild CM16

C. medica Tinsukia-Assam Wild CM17

C. medica Tinsukia-Assam Wild CM18

C. medica Tinsukia-Assam Wild CM19

C. medica Tinsukia-Assam Wild CM20

C. medica Banskandi-Assam Domestic CM21

C. medica Banskandi-Assam Domestic CM22

C. medica Banskandi-Assam Domestic CM23

C. medica Banskandi-Assam Domestic CM24

C. medica Banskandi-Assam Domestic CM25

C. medica Banskandi-Assam Domestic CM26

C. medica Banskandi-Assam Domestic CM27

C. medica Banskandi-Assam Domestic CM28

C. medica Banskandi-Assam Domestic CM29

C. medica Banskandi-Assam Domestic CM30

C. medica Banskandi-Assam Domestic CM31

C. medica Banskandi-Assam Domestic CM32

C. medica Banskandi-Assam Domestic CM33

C. medica Banskandi-Assam Domestic CM34

C. medica Banskandi-Assam Domestic CM35

C. medica Banskandi-Assam Domestic CM36

C. medica Banskandi-Assam Domestic CM37

C. medica Banskandi-Assam Domestic CM38

C. medica Banskandi-Assam Domestic CM39

C. medica Banskandi-Assam Domestic CM40

C. medica Itanagar-Arunachal Pradesh Domestic CM41

C. medica Itanagar-Arunachal Pradesh Domestic CM42

C. medica Itanagar-Arunachal Pradesh Domestic CM43

C. medica Itanagar-Arunachal Pradesh Domestic CM44

C. medica Itanagar-Arunachal Pradesh Domestic CM45

C. medica Itanagar-Arunachal Pradesh Domestic CM46

C. medica Itanagar-Arunachal Pradesh Domestic CM47

C. medica Itanagar-Arunachal Pradesh Domestic CM48

C. medica Itanagar-Arunachal Pradesh Domestic CM49

C. medica Itanagar-Arunachal Pradesh Domestic CM50

C. medica Itanagar-Arunachal Pradesh Domestic CM51

C. medica Itanagar-Arunachal Pradesh Domestic CM52

C. medica Itanagar-Arunachal Pradesh Domestic CM53

C. medica Itanagar-Arunachal Pradesh Domestic CM54

C. medica Itanagar-Arunachal Pradesh Domestic CM55

C. medica Itanagar-Arunachal Pradesh Domestic CM56

C. medica Itanagar-Arunachal Pradesh Domestic CM57

C. medica Itanagar-Arunachal Pradesh Domestic CM58

C. medica Itanagar-Arunachal Pradesh Domestic CM59

C. medica Itanagar-Arunachal Pradesh Domestic CM60

C. medica Aizawl-Mizoram Domestic CM61

C. medica Aizawl-Mizoram Domestic CM62

C. medica Aizawl-Mizoram Domestic CM63

C. medica Aizawl-Mizoram Domestic CM64

C. medica Aizawl-Mizoram Domestic CM65

C. medica Aizawl-Mizoram Domestic CM66

C. medica Aizawl-Mizoram Domestic CM67

C. medica Aizawl-Mizoram Domestic CM68

C. medica Aizawl-Mizoram Domestic CM69

C. medica Aizawl-Mizoram Domestic CM70

C. medica Aizawl-Mizoram Domestic CM71

C. medica Aizawl-Mizoram Domestic CM72

C. medica Aizawl-Mizoram Domestic CM73

C. medica Aizawl-Mizoram Domestic CM74

C. medica Aizawl-Mizoram Domestic CM75

C. medica Aizawl-Mizoram Domestic CM76

C. medica Aizawl-Mizoram Domestic CM77

C. medica Aizawl-Mizoram Domestic CM78

C. medica Aizawl-Mizoram Domestic CM79

C. medica Aizawl-Mizoram Domestic CM80

C. medica Sairang1-Mizoram Wild CM81

C. medica Sairang1-Mizoram Wild CM82

C. medica Sairang1-Mizoram Wild CM83

C. medica Sairang1-Mizoram Wild CM84

C. medica Sairang1-Mizoram Wild CM85

C. medica Sairang1-Mizoram Wild CM86

C. medica Sairang1-Mizoram Wild CM87

C. medica Sairang1-Mizoram Wild CM88

C. medica Sairang1-Mizoram Wild CM89

C. medica Sairang1-Mizoram Wild CM90

C. medica Sairang1-Mizoram Wild CM91

C. medica Sairang1-Mizoram Wild CM92

C. medica Sairang1-Mizoram Wild CM93

C. medica Sairang1-Mizoram Wild CM94

C. medica Sairang1-Mizoram Wild CM95

C. medica Sairang1-Mizoram Wild CM96

C. medica Sairang1-Mizoram Wild CM97

C. medica Sairang1-Mizoram Wild CM98

C. medica Sairang1-Mizoram Wild CM99

C. medica Sairang1-Mizoram Wild CM100

C. medica Sairang2-Mizoram Domestic CM101

C. medica Sairang2-Mizoram Domestic CM102

C. medica Sairang2-Mizoram Domestic CM103

C. medica Sairang2-Mizoram Domestic CM104

C. medica Sairang2-Mizoram Domestic CM105

C. medica Sairang2-Mizoram Domestic CM106

C. medica Sairang2-Mizoram Domestic CM107

C. medica Sairang2-Mizoram Domestic CM108

C. medica Sairang2-Mizoram Domestic CM109

C. medica Sairang2-Mizoram Domestic CM110

C. medica Sairang2-Mizoram Domestic CM111

C. medica Sairang2-Mizoram Domestic CM112

C. medica Sairang2-Mizoram Domestic CM113

C. medica Sairang2-Mizoram Domestic CM114

C. medica Sairang2-Mizoram Domestic CM115

C. medica Sairang2-Mizoram Domestic CM116

C. medica Sairang2-Mizoram Domestic CM117

C. medica Sairang2-Mizoram Domestic CM118

C. medica Sairang2-Mizoram Domestic CM119

C. medica Sairang2-Mizoram Domestic CM120

C. medica Motinagar1-Assam Domestic CM121

C. medica Motinagar1-Assam Domestic CM122

C. medica Motinagar1-Assam Domestic CM123

C. medica Motinagar1-Assam Domestic CM124

C. medica Motinagar1-Assam Domestic CM125

C. medica Motinagar1-Assam Domestic CM126

C. medica Motinagar1-Assam Domestic CM127

C. medica Motinagar1-Assam Domestic CM128

C. medica Motinagar1-Assam Domestic CM129

C. medica Motinagar1-Assam Domestic CM130

C. medica Motinagar1-Assam Domestic CM131

C. medica Motinagar1-Assam Domestic CM132

C. medica Motinagar1-Assam Domestic CM133

C. medica Motinagar1-Assam Domestic CM134

C. medica Motinagar1-Assam Domestic CM135

C. medica Motinagar1-Assam Domestic CM136

C. medica Motinagar1-Assam Domestic CM137

C. medica Motinagar1-Assam Domestic CM138

C. medica Motinagar1-Assam Domestic CM139

C. medica Motinagar1-Assam Domestic CM140

C. medica Motinagar2-Assam Wild CM141

C. medica Motinagar2-Assam Wild CM142

C. medica Motinagar2-Assam Wild CM143

C. medica Motinagar2-Assam Wild CM144

C. medica Motinagar2-Assam Wild CM145

C. medica Motinagar2-Assam Wild CM146

C. medica Motinagar2-Assam Wild CM147

C. medica Motinagar2-Assam Wild CM148

C. medica Motinagar2-Assam Wild CM149

C. medica Motinagar2-Assam Wild CM150

C. medica Motinagar2-Assam Wild CM151

C. medica Motinagar2-Assam Wild CM152

C. medica Motinagar2-Assam Wild CM153

C. medica Motinagar2-Assam Wild CM154

C. medica Motinagar2-Assam Wild CM155

C. medica Motinagar2-Assam Wild CM156

C. medica Motinagar2-Assam Wild CM157

C. medica Motinagar2-Assam Wild CM158

C. medica Motinagar2-Assam Wild CM159

C. medica Motinagar2-Assam Wild CM160

C. medica Lakhipur-Assam Domestic CM161

C. medica Lakhipur-Assam Domestic CM162

C. medica Lakhipur-Assam Domestic CM163

C. medica Lakhipur-Assam Domestic CM164

C. medica Lakhipur-Assam Domestic CM165

C. medica Lakhipur-Assam Domestic CM166

C. medica Lakhipur-Assam Domestic CM167

C. medica Lakhipur-Assam Domestic CM168

C. medica Lakhipur-Assam Domestic CM169

C. medica Lakhipur-Assam Domestic CM170

C. medica Lakhipur-Assam Domestic CM171

C. medica Lakhipur-Assam Domestic CM172

C. medica Lakhipur-Assam Domestic CM173

C. medica Lakhipur-Assam Domestic CM174

C. medica Lakhipur-Assam Domestic CM175

C. medica Lakhipur-Assam Domestic CM176

C. medica Lakhipur-Assam Domestic CM177

C. medica Lakhipur-Assam Domestic CM178

C. medica Lakhipur-Assam Domestic CM179

C. medica Lakhipur-Assam Domestic CM180

C. medica Sonai-Assam Domestic CM181

C. medica Sonai-Assam Domestic CM182

C. medica Sonai-Assam Domestic CM183

C. medica Sonai-Assam Domestic CM184

C. medica Sonai-Assam Domestic CM185

C. medica Sonai-Assam Domestic CM186

C. medica Sonai-Assam Domestic CM187

C. medica Sonai-Assam Domestic CM188

C. medica Sonai-Assam Domestic CM189

C. medica Sonai-Assam Domestic CM190

C. medica Sonai-Assam Domestic CM191

C. medica Sonai-Assam Domestic CM192

C. medica Sonai-Assam Domestic CM193

C. medica Sonai-Assam Domestic CM194

C. medica Sonai-Assam Domestic CM195

C. medica Sonai-Assam Domestic CM196

C. medica Sonai-Assam Domestic CM197

C. medica Sonai-Assam Domestic CM198

C. medica Sonai-Assam Domestic CM199

C. medica Sonai-Assam Domestic CM200

C. medica Neairgram-Assam Domestic CM201

C. medica Neairgram-Assam Domestic CM202

C. medica Neairgram-Assam Domestic CM203

C. medica Neairgram-Assam Domestic CM204

C. medica Neairgram-Assam Domestic CM205

C. medica Neairgram-Assam Domestic CM206

C. medica Neairgram-Assam Domestic CM207

C. medica Neairgram-Assam Domestic CM208

C. medica Neairgram-Assam Domestic CM209

C. medica Neairgram-Assam Domestic CM210

C. medica Neairgram-Assam Domestic CM211

C. medica Neairgram-Assam Domestic CM212

C. medica Neairgram-Assam Domestic CM213

C. medica Neairgram-Assam Domestic CM214

C. medica Neairgram-Assam Domestic CM215

C. medica Namsai-Arunachal Pradesh Wild CM216

C. medica Namsai-Arunachal Pradesh Wild M217

C. medica Namsai-Arunachal Pradesh Wild CM218

C. medica Namsai-Arunachal Pradesh Wild CM219

**Supplementary Table 2. Genotypes of *Citrus medica* population in NE India.**

(Number of populations 12 and number of loci 5)

SSR03 SSR04 SSR05 SSR06 SSR07

pop = 1Tinsukia_Assam_Wild

CM1 188 188 245 245 225 225 199 199 193 193

CM2 243 243 242 245 227 227 199 199 193 193

CM3 243 243 239 239 225 225 199 199 193 193

CM4 240 240 242 242 231 233 198 198 193 193

CM5 188 188 242 242 222 225 198 198 193 193

CM6 188 189 245 245 222 225 198 198 192 192

CM7 243 243 243 243 231 225 198 198 192 192

CM8 240 243 239 239 226 226 198 198 192 192

CM9 240 240 242 242 224 224 198 198 192 192

CM10 188 189 239 239 222 226 198 198 192 192

CM11 188 189 239 239 231 234 198 198 195 195

CM12 240 240 239 243 234 234 198 198 195 195

CM13 186 188 243 243 231 234 197 197 195 195

CM14 188 188 239 239 231 231 197 197 195 195

CM15 182 186 239 239 231 231 198 198 195 195

CM16 188 188 239 239 231 231 194 197 195 213

CM17 191 191 239 242 230 233 194 197 195 195

CM18 191 191 239 243 230 233 194 194 195 195

CM19 188 188 242 242 233 233 197 197 195 195

CM20 188 188 242 242 233 233 198 198 195 195

pop = 2Banskandi_Assam

CM21 186 188 239 242 220 224 198 198 192 192

CM22 186 188 240 243 224 224 198 198 192 192

CM23 240 240 240 240 224 224 198 198 192 192

CM24 186 188 242 242 224 225 198 199 192 206

CM25 186 188 239 242 222 226 198 198 192 192

CM26 186 188 239 242 223 223 198 198 192 192

CM27 186 188 239 242 223 225 198 198 192 192

CM28 186 188 242 242 223 225 198 199 192 192

CM29 186 188 242 244 223 225 198 199 192 192

CM30 188 190 242 244 223 225 198 198 192 201

CM31 188 194 239 242 233 233 196 196 195 195

CM32 188 187 242 242 227 233 196 196 195 195

CM33 188 187 239 242 232 233 196 198 195 195

CM34 191 191 239 242 231 232 196 198 195 195

CM35 182 188 242 242 229 231 195 195 195 195

CM36 188 188 239 242 231 231 195 197 195 212

CM37 186 188 242 243 231 233 195 195 195 195

CM38 186 188 243 243 230 231 195 197 195 195

CM39 188 188 242 242 231 233 195 195 195 207

CM40 186 186 239 242 231 233 195 195 195 195

pop = 3Itanagar_AP

CM41 188 188 239 242 224 226 199 199 192 201

CM42 188 188 239 239 226 226 199 199 192 201

CM43 188 189 243 243 226 226 199 199 192 192

CM44 188 189 239 242 225 226 199 199 192 192

CM45 188 189 242 242 226 226 199 199 192 201

CM46 188 189 242 243 223 227 199 199 195 195

CM47 188 189 238 242 225 225 199 199 195 206

CM48 188 189 239 243 223 227 199 199 204 206

CM49 188 188 239 239 225 225 199 199 195 206

CM50 188 189 242 242 224 224 199 199 195 195

CM51 243 243 239 239 231 231 195 195 203 203

CM52 191 191 239 242 231 233 195 195 195 195

CM53 243 246 239 239 225 231 195 195 195 195

CM54 188 188 239 239 225 231 195 195 195 195

CM55 188 188 239 239 228 231 195 195 195 195

CM56 188 188 239 239 230 231 195 195 195 195

CM57 186 188 239 242 230 131 195 195 195 195

CM58 243 246 242 242 230 231 195 195 195 202

CM59 240 243 239 242 230 231 195 195 195 195

CM60 188 188 242 242 230 231 195 195 195 195

pop = 4Mizo_Aizawl

CM61 188 189 242 242 225 225 199 199 195 195

CM62 188 188 239 239 228 230 199 199 195 195

CM63 188 188 243 242 225 225 199 199 195 195

CM64 188 189 239 243 223 225 199 199 195 195

CM65 186 189 239 239 223 225 199 199 195 215

CM66 188 188 239 242 225 225 199 199 195 215

CM67 186 189 239 243 225 225 198 199 195 210

CM68 186 189 242 242 226 227 199 199 195 206

CM69 186 186 239 243 225 227 199 199 195 195

CM70 189 186 242 243 222 226 199 199 195 206

CM71 186 186 239 242 228 231 195 195 195 195

CM72 186 186 239 242 230 231 195 195 195 195

CM73 188 188 239 242 225 231 195 195 195 195

CM74 188 188 241 242 230 231 195 195 195 195

CM75 182 188 239 243 230 231 195 195 195 195

CM76 188 188 240 240 229 232 195 195 195 195

CM77 188 188 239 243 225 231 195 195 195 195

CM78 188 188 239 243 229 231 195 195 195 195

CM79 188 188 239 243 225 231 195 195 195 201

CM80 240 243 242 239 225 231 193 195 195 195

pop = 5Mizo_Sairang

CM81 189 189 242 239 233 233 199 199 196 196

CM82 243 243 242 239 233 231 199 199 216 196

CM83 189 189 239 239 233 233 199 200 196 196

CM84 189 189 242 239 233 233 199 199 208 196

CM85 188 188 242 239 235 234 199 199 204 196

CM86 240 243 242 242 235 233 200 199 196 196

CM87 188 188 239 239 235 131 200 199 204 196

CM88 188 188 242 239 233 233 199 199 208 196

CM89 243 243 242 239 221 225 197 198 195 195

CM90 188 188 242 239 235 233 200 199 196 196

CM91 240 243 242 239 233 228 199 199 204 196

CM92 188 188 240 240 233 233 199 199 196 196

CM93 188 188 240 242 235 233 200 199 196 196

CM94 194 194 240 242 233 233 199 199 204 196

CM95 188 188 242 242 233 233 199 199 204 196

CM96 194 194 243 243 230 231 199 199 196 196

CM97 194 194 239 240 233 233 199 199 196 196

CM98 188 188 239 239 233 233 199 199 206 196

CM99 194 194 242 242 233 233 199 199 196 196

CM100 182 188 242 242 233 235 200 199 196 196

pop = 6Mizo_Sairang_Wild

CM101 188 188 242 239 231 231 199 199 196 196

CM102 188 188 239 239 233 231 199 199 196 196

CM103 188 188 240 240 226 231 199 199 204 196

CM104 188 188 239 239 235 233 199 199 196 196

CM105 190 190 240 240 233 233 200 199 196 196

CM106 188 188 242 242 235 233 200 199 196 196

CM107 188 188 242 242 233 228 199 199 196 196

CM108 188 188 242 242 235 233 200 199 196 196

CM109 188 188 239 239 233 235 199 199 196 196

CM110 194 194 242 239 233 235 199 199 208 196

CM111 188 188 242 242 233 233 196 196 208 206

CM112 188 188 242 240 233 233 199 199 196 196

CM113 188 188 239 239 234 231 199 199 196 196

CM114 188 188 242 242 235 233 199 199 196 196

CM115 194 194 242 239 235 233 199 199 196 196

CM116 188 188 239 239 235 234 199 199 204 196

CM117 188 188 242 239 235 231 199 199 198 196

CM118 188 194 242 239 233 231 199 199 196 196

CM119 188 188 239 239 233 231 199 199 196 196

CM120 188 194 242 239 235 231 199 199 196 196

pop = 7Motinagar_assam

CM121 187 190 239 239 228 228 199 199 196 196

CM122 188 190 242 239 235 231 199 199 196 196

CM123 188 188 239 239 235 233 199 199 196 196

CM124 188 188 239 239 135 131 199 199 196 196

CM125 188 188 242 239 134 134 199 199 196 196

CM126 188 188 239 239 134 133 199 199 196 196

CM127 188 188 242 239 134 131 199 199 196 196

CM128 188 188 239 239 134 133 199 199 196 196

CM129 188 194 239 239 133 131 199 199 196 196

CM130 188 188 242 242 135 131 199 199 196 196

CM131 188 188 240 240 233 233 197 197 196 196

CM132 188 194 239 239 131 131 199 199 196 196

CM133 187 187 243 243 233 233 196 196 195 195

CM134 188 190 242 242 233 233 196 196 195 195

CM135 188 190 242 242 233 233 196 196 196 196

CM136 188 190 242 242 228 228 200 200 195 195

CM137 188 190 242 239 233 233 196 196 204 196

CM138 ? ? 239 239 232 234 196 200 196 196

CM139 ? ? 243 245 232 232 196 196 195 195

CM140 188 188 242 239 133 131 199 199 194 194

pop = 8Assam_Motinagar_Wild

CM141 189 189 240 240 233 233 197 198 194 194

CM142 187 187 244 244 228 228 196 197 194 194

CM143 189 189 244 244 233 233 196 196 196 215

CM144 189 189 244 244 233 233 196 198 196 196

CM145 189 189 244 244 232 232 196 197 194 194

CM146 189 189 240 240 232 232 196 197 196 196

CM147 189 189 240 240 232 232 196 197 194 194

CM148 189 189 240 240 233 233 196 199 196 196

CM149 190 190 240 240 233 233 196 199 194 194

CM150 189 189 243 243 233 233 195 197 194 194

CM151 189 189 240 243 232 232 195 196 194 194

CM152 189 194 240 240 232 232 195 196 194 194

CM153 189 194 240 240 233 233 195 198 196 207

CM154 189 194 243 245 233 233 195 196 194 194

CM155 189 194 240 243 ? ? 196 198 195 195

CM156 189 194 240 240 230 232 195 196 195 195

CM157 189 194 240 240 232 232 195 197 196 207

CM158 189 189 240 240 232 232 195 196 196 207

CM159 189 189 240 240 230 232 195 198 195 195

CM160 189 194 244 244 228 233 195 196 196 207

pop = 9Lakhipur_Assam

CM161 189 194 240 244 228 233 195 196 195 195

CM162 189 189 244 244 228 228 196 196 196 207

CM163 189 189 240 244 223 228 195 196 195 195

CM164 188 190 240 244 223 228 195 196 196 208

CM165 189 189 240 244 228 233 197 197 195 195

CM166 189 189 240 245 232 232 195 196 196 196

CM167 189 194 240 240 233 234 195 196 196 207

CM168 189 189 240 240 229 229 195 196 195 195

CM169 189 189 240 240 233 235 196 196 195 195

CM170 189 189 240 240 233 233 196 196 195 195

CM171 189 189 240 243 232 233 196 196 195 195

CM172 189 194 243 244 233 233 195 196 195 195

CM173 189 189 243 243 232 232 195 196 195 195

CM174 189 189 240 240 233 233 195 196 195 195

CM175 189 194 243 248 230 233 195 196 195 195

CM176 189 189 243 243 233 233 195 196 195 195

CM177 189 194 243 248 233 234 195 196 195 195

CM178 189 189 240 244 232 232 196 196 195 195

CM179 189 189 240 244 233 233 196 196 195 195

CM180 189 189 240 240 233 233 196 196 195 195

pop = 10Sonai_Assam

CM181 189 194 243 243 230 233 195 196 195 195

CM182 189 189 243 243 230 233 195 196 195 195

CM183 189 189 240 243 228 228 196 196 195 195

CM184 189 194 240 240 228 228 196 196 195 195

CM185 189 189 240 240 233 233 196 196 195 195

CM186 189 194 243 248 233 233 197 197 195 195

CM187 189 189 243 243 233 233 196 197 195 195

CM188 189 189 243 243 230 233 195 196 195 195

CM189 243 244 240 243 233 233 198 199 193 195

CM190 240 240 240 240 233 233 198 199 195 195

CM191 240 243 240 243 233 233 198 198 195 195

CM192 240 244 240 244 233 234 198 198 195 195

CM193 243 243 243 243 228 228 196 198 195 195

CM194 243 243 240 240 228 228 197 200 195 195

CM195 240 240 243 244 228 228 195 196 195 195

CM196 240 240 240 243 233 233 196 197 195 195

CM197 243 244 240 240 233 233 196 197 195 195

CM198 240 240 240 240 231 233 198 198 195 195

CM199 240 240 240 243 233 233 197 198 193 193

CM200 243 244 240 243 233 233 196 200 193 193

pop = 11Neairgram_Assam

CM201 243 244 240 243 232 233 197 198 193 193

CM202 240 240 243 243 231 233 197 198 193 193

CM203 243 243 240 241 231 233 197 198 193 193

CM204 240 240 243 243 234 235 197 198 193 193

CM205 243 243 240 240 233 233 197 198 193 193

CM206 240 244 240 241 229 233 197 198 193 193

CM207 243 243 240 240 234 235 197 197 195 195

CM208 243 243 240 243 229 229 197 198 193 193

CM209 243 244 243 243 229 233 197 198 195 195

CM210 240 240 240 243 233 233 196 200 195 195

CM211 243 244 240 240 233 233 197 198 193 193

CM212 243 244 240 245 233 233 197 198 193 193

CM213 243 243 243 243 229 233 197 198 195 195

CM214 240 240 243 243 229 233 197 198 195 195

CM215 240 240 243 245 232 232 197 197 193 193

pop = 12Namsai_AP_wild

CM216 240 240 240 240 233 233 197 198 195 195

CM217 243 243 243 244 233 233 197 198 195 215

CM218 240 244 240 243 232 234 197 198 195 195

CM219 240 240 240 240 233 233 197 198 195 195

**Supplementary Table 3. Allele frequency comparison over populations**

Key to Population Names:

~~~~~~~~~~~~~~~~~~~~~~~~

Pop1 1Tinsukia_Assam_Wild

Pop2 2Banskandi_Assam

Pop3 3Itanagar_AP

Pop4 4Mizo_Aizawl

Pop5 5Mizo_Sairang

Pop6 6Mizo_Sairang_Wild

Pop7 7Motinagar_assam

Pop8 8Assam_Motinagar_Wild

Pop9 9Lakhipur_Assam

Pop10 10Sonai_Assam

Pop11 11Neairgram_Assam

Pop12 12Namsai_AP_wild

Locus Allele# Size Pop1 Pop2 Pop3 Pop4 Pop5 Pop6 Pop7 Pop8 Pop9 Pop10 Pop11 Pop12 Overall Private?

-------- ------- ---- ------ ------ ------ ------ ------ ------ ------ ------ ------ ------ ------ ------ ------- --------

SSR03 1 182 0.0250 0.0250 0.0000 0.0250 0.0250 0.0000 0.0000 0.0000 0.0000 0.0000 0.0000 0.0000 0.0092

SSR03 2 186 0.0500 0.3000 0.0250 0.2500 0.0000 0.0000 0.0000 0.0000 0.0000 0.0000 0.0000 0.0000 0.0576

SSR03 3 187 0.0000 0.0500 0.0000 0.0000 0.0000 0.0000 0.0833 0.0500 0.0000 0.0000 0.0000 0.0000 0.0161

SSR03 4 188 0.4000 0.4750 0.5500 0.5250 0.4250 0.8000 0.6944 0.0000 0.0250 0.0000 0.0000 0.0000 0.3525

SSR03 5 189 0.0750 0.0000 0.1750 0.1500 0.1500 0.0000 0.0000 0.7250 0.8250 0.3250 0.0000 0.0000 0.2235

SSR03 6 190 0.0000 0.0250 0.0000 0.0000 0.0000 0.0500 0.1667 0.0500 0.0250 0.0000 0.0000 0.0000 0.0276

SSR03 7 191 0.1000 0.0500 0.0500 0.0000 0.0000 0.0000 0.0000 0.0000 0.0000 0.0000 0.0000 0.0000 0.0184

SSR03 8 194 0.0000 0.0250 0.0000 0.0000 0.2000 0.1500 0.0556 0.1750 0.1250 0.0750 0.0000 0.0000 0.0737

SSR03 9 240 0.1750 0.0500 0.0250 0.0250 0.0500 0.0000 0.0000 0.0000 0.0000 0.3000 0.3667 0.6250 0.0945

SSR03 10 243 0.1750 0.0000 0.1250 0.0250 0.1500 0.0000 0.0000 0.0000 0.0000 0.2000 0.4667 0.2500 0.0991

SSR03 11 244 0.0000 0.0000 0.0000 0.0000 0.0000 0.0000 0.0000 0.0000 0.0000 0.1000 0.1667 0.1250 0.0230

SSR03 12 246 0.0000 0.0000 0.0500 0.0000 0.0000 0.0000 0.0000 0.0000 0.0000 0.0000 0.0000 0.0000 0.0046 3Itanagar_AP

SSR03 # samples: 20 20 20 20 20 20 18 20 20 20 15 4 217

Locus Allele# Size Pop1 Pop2 Pop3 Pop4 Pop5 Pop6 Pop7 Pop8 Pop9 Pop10 Pop11 Pop12 Overall Private?

-------- ------- ---- ------ ------ ------ ------ ------ ------ ------ ------ ------ ------ ------ ------ ------- --------

SSR04 1 238 0.0000 0.0000 0.0250 0.0000 0.0000 0.0000 0.0000 0.0000 0.0000 0.0000 0.0000 0.0000 0.0023 3Itanagar_AP

SSR04 2 239 0.4250 0.2250 0.5000 0.4000 0.3750 0.4500 0.5250 0.0000 0.0000 0.0000 0.0000 0.0000 0.2648

SSR04 3 240 0.0000 0.0750 0.0000 0.0500 0.1250 0.1250 0.0500 0.6000 0.5000 0.4750 0.4000 0.6250 0.2215

SSR04 4 241 0.0000 0.0000 0.0000 0.0250 0.0000 0.0000 0.0000 0.0000 0.0000 0.0000 0.0667 0.0000 0.0068

SSR04 5 242 0.3000 0.5500 0.3750 0.3000 0.4500 0.4250 0.3250 0.0000 0.0000 0.0000 0.0000 0.0000 0.2489

SSR04 6 243 0.1500 0.1000 0.1000 0.2250 0.0500 0.0000 0.0750 0.1250 0.2000 0.4500 0.4667 0.2500 0.1712

SSR04 7 244 0.0000 0.0500 0.0000 0.0000 0.0000 0.0000 0.0000 0.2500 0.2250 0.0500 0.0000 0.1250 0.0548

SSR04 8 245 0.1250 0.0000 0.0000 0.0000 0.0000 0.0000 0.0250 0.0250 0.0250 0.0000 0.0667 0.0000 0.0228

SSR04 9 248 0.0000 0.0000 0.0000 0.0000 0.0000 0.0000 0.0000 0.0000 0.0500 0.0250 0.0000 0.0000 0.0068

SSR04 # samples: 20 20 20 20 20 20 20 20 20 20 15 4 219

Locus Allele# Size Pop1 Pop2 Pop3 Pop4 Pop5 Pop6 Pop7 Pop8 Pop9 Pop10 Pop11 Pop12 Overall Private?

-------- ------- ---- ------ ------ ------ ------ ------ ------ ------ ------ ------ ------ ------ ------ ------- --------

SSR05 1 131 0.0000 0.0000 0.0250 0.0000 0.0250 0.0000 0.1750 0.0000 0.0000 0.0000 0.0000 0.0000 0.0206

SSR05 2 133 0.0000 0.0000 0.0000 0.0000 0.0000 0.0000 0.1000 0.0000 0.0000 0.0000 0.0000 0.0000 0.0092 7Motinagar_assam

SSR05 3 134 0.0000 0.0000 0.0000 0.0000 0.0000 0.0000 0.1250 0.0000 0.0000 0.0000 0.0000 0.0000 0.0115 7Motinagar_assam

SSR05 4 135 0.0000 0.0000 0.0000 0.0000 0.0000 0.0000 0.0500 0.0000 0.0000 0.0000 0.0000 0.0000 0.0046 7Motinagar_assam

SSR05 5 220 0.0000 0.0250 0.0000 0.0000 0.0000 0.0000 0.0000 0.0000 0.0000 0.0000 0.0000 0.0000 0.0023 2Banskandi_Assam

SSR05 6 221 0.0000 0.0000 0.0000 0.0000 0.0250 0.0000 0.0000 0.0000 0.0000 0.0000 0.0000 0.0000 0.0023 5Mizo_Sairang

SSR05 7 222 0.0750 0.0250 0.0000 0.0250 0.0000 0.0000 0.0000 0.0000 0.0000 0.0000 0.0000 0.0000 0.0115

SSR05 8 223 0.0000 0.1500 0.0500 0.0500 0.0000 0.0000 0.0000 0.0000 0.0500 0.0000 0.0000 0.0000 0.0275

SSR05 9 224 0.0500 0.1500 0.0750 0.0000 0.0000 0.0000 0.0000 0.0000 0.0000 0.0000 0.0000 0.0000 0.0252

SSR05 10 225 0.1750 0.1250 0.1750 0.3750 0.0250 0.0000 0.0000 0.0000 0.0000 0.0000 0.0000 0.0000 0.0803

SSR05 11 226 0.0750 0.0250 0.2000 0.0500 0.0000 0.0250 0.0000 0.0000 0.0000 0.0000 0.0000 0.0000 0.0344

SSR05 12 227 0.0500 0.0250 0.0500 0.0500 0.0000 0.0000 0.0000 0.0000 0.0000 0.0000 0.0000 0.0000 0.0161

SSR05 13 228 0.0000 0.0000 0.0250 0.0500 0.0250 0.0250 0.1000 0.0789 0.1500 0.2500 0.0000 0.0000 0.0642

SSR05 14 229 0.0000 0.0250 0.0000 0.0500 0.0000 0.0000 0.0000 0.0000 0.0500 0.0000 0.2000 0.0000 0.0252

SSR05 15 230 0.0500 0.0250 0.1250 0.1000 0.0250 0.0000 0.0000 0.0526 0.0250 0.0750 0.0000 0.0000 0.0436

SSR05 16 231 0.2500 0.2000 0.2500 0.2250 0.0500 0.2250 0.0250 0.0000 0.0000 0.0250 0.0667 0.0000 0.1193

SSR05 17 232 0.0000 0.0500 0.0000 0.0250 0.0000 0.0000 0.0750 0.4211 0.1750 0.0000 0.1000 0.1250 0.0757

SSR05 18 233 0.1750 0.1750 0.0250 0.0000 0.6500 0.4250 0.2750 0.4474 0.4750 0.6250 0.5000 0.7500 0.3463

SSR05 19 234 0.1000 0.0000 0.0000 0.0000 0.0250 0.0500 0.0250 0.0000 0.0500 0.0250 0.0667 0.1250 0.0321

SSR05 20 235 0.0000 0.0000 0.0000 0.0000 0.1500 0.2500 0.0500 0.0000 0.0250 0.0000 0.0667 0.0000 0.0482

SSR05 # samples: 20 20 20 20 20 20 20 19 20 20 15 4 218

Locus Allele# Size Pop1 Pop2 Pop3 Pop4 Pop5 Pop6 Pop7 Pop8 Pop9 Pop10 Pop11 Pop12 Overall Private?

-------- ------- ---- ------ ------ ------ ------ ------ ------ ------ ------ ------ ------ ------ ------ ------- --------

SSR06 1 193 0.0000 0.0000 0.0000 0.0250 0.0000 0.0000 0.0000 0.0000 0.0000 0.0000 0.0000 0.0000 0.0023 4Mizo_Aizawl

SSR06 2 194 0.1000 0.0000 0.0000 0.0000 0.0000 0.0000 0.0000 0.0000 0.0000 0.0000 0.0000 0.0000 0.0091 1Tinsukia_Assam_Wild

SSR06 3 195 0.0000 0.2500 0.5000 0.4750 0.0000 0.0000 0.0000 0.2500 0.3000 0.1000 0.0000 0.0000 0.1712

SSR06 4 196 0.0000 0.1500 0.0000 0.0000 0.0000 0.0500 0.2750 0.4000 0.6500 0.3750 0.0333 0.0000 0.1758

SSR06 5 197 0.2000 0.0500 0.0000 0.0000 0.0250 0.0000 0.0500 0.1750 0.0500 0.1750 0.5333 0.5000 0.1119

SSR06 6 198 0.5500 0.4750 0.0000 0.0250 0.0250 0.0000 0.0000 0.1250 0.0000 0.2500 0.4000 0.5000 0.1689

SSR06 7 199 0.1500 0.0750 0.5000 0.4750 0.8000 0.8750 0.6000 0.0500 0.0000 0.0500 0.0000 0.0000 0.3265

SSR06 8 200 0.0000 0.0000 0.0000 0.0000 0.1500 0.0750 0.0750 0.0000 0.0000 0.0500 0.0333 0.0000 0.0342

SSR06 # samples: 20 20 20 20 20 20 20 20 20 20 15 4 219

Locus Allele# Size Pop1 Pop2 Pop3 Pop4 Pop5 Pop6 Pop7 Pop8 Pop9 Pop10 Pop11 Pop12 Overall Private?

-------- ------- ---- ------ ------ ------ ------ ------ ------ ------ ------ ------ ------ ------ ------ ------- --------

SSR07 1 192 0.2500 0.4500 0.1750 0.0000 0.0000 0.0000 0.0000 0.0000 0.0000 0.0000 0.0000 0.0000 0.0799

SSR07 2 193 0.2500 0.0000 0.0000 0.0000 0.0000 0.0000 0.0000 0.0000 0.0000 0.1250 0.6667 0.0000 0.0799

SSR07 3 194 0.0000 0.0000 0.0000 0.0000 0.0000 0.0000 0.0500 0.4500 0.0000 0.0000 0.0000 0.0000 0.0457

SSR07 4 195 0.4750 0.4500 0.5750 0.8500 0.0500 0.0000 0.2000 0.1500 0.8000 0.8750 0.3333 0.8750 0.4429

SSR07 5 196 0.0000 0.0000 0.0000 0.0000 0.7250 0.8500 0.7250 0.2750 0.1250 0.0000 0.0000 0.0000 0.2466

SSR07 6 198 0.0000 0.0000 0.0000 0.0000 0.0000 0.0250 0.0000 0.0000 0.0000 0.0000 0.0000 0.0000 0.0023 6Mizo_Sairang_Wild

SSR07 7 201 0.0000 0.0250 0.0750 0.0250 0.0000 0.0000 0.0000 0.0000 0.0000 0.0000 0.0000 0.0000 0.0114

SSR07 8 202 0.0000 0.0000 0.0250 0.0000 0.0000 0.0000 0.0000 0.0000 0.0000 0.0000 0.0000 0.0000 0.0023 3Itanagar_AP

SSR07 9 203 0.0000 0.0000 0.0500 0.0000 0.0000 0.0000 0.0000 0.0000 0.0000 0.0000 0.0000 0.0000 0.0046 3Itanagar_AP

SSR07 10 204 0.0000 0.0000 0.0250 0.0000 0.1250 0.0500 0.0250 0.0000 0.0000 0.0000 0.0000 0.0000 0.0205

SSR07 11 206 0.0000 0.0250 0.0750 0.0500 0.0250 0.0250 0.0000 0.0000 0.0000 0.0000 0.0000 0.0000 0.0183

SSR07 12 207 0.0000 0.0250 0.0000 0.0000 0.0000 0.0000 0.0000 0.1000 0.0500 0.0000 0.0000 0.0000 0.0160

SSR07 13 208 0.0000 0.0000 0.0000 0.0000 0.0500 0.0500 0.0000 0.0000 0.0250 0.0000 0.0000 0.0000 0.0114

SSR07 14 210 0.0000 0.0000 0.0000 0.0250 0.0000 0.0000 0.0000 0.0000 0.0000 0.0000 0.0000 0.0000 0.0023 4Mizo_Aizawl

SSR07 15 212 0.0000 0.0250 0.0000 0.0000 0.0000 0.0000 0.0000 0.0000 0.0000 0.0000 0.0000 0.0000 0.0023 2Banskandi_Assam

SSR07 16 213 0.0250 0.0000 0.0000 0.0000 0.0000 0.0000 0.0000 0.0000 0.0000 0.0000 0.0000 0.0000 0.0023 1Tinsukia_Assam_Wild

SSR07 17 215 0.0000 0.0000 0.0000 0.0500 0.0000 0.0000 0.0000 0.0250 0.0000 0.0000 0.0000 0.1250 0.0091

SSR07 18 216 0.0000 0.0000 0.0000 0.0000 0.0250 0.0000 0.0000 0.0000 0.0000 0.0000 0.0000 0.0000 0.0023 5Mizo_Sairang

SSR07 # samples: 20 20 20 20 20 20 20 20 20 20 15 4 219

**Supplementary Figure 1.** Relationship between geographic distance and Nei’s genetic distance among the twelve populations of wild and domestic *C. medica*.

**Supplementary Figure 2.** Relationship between geographic distance and genetic differentiation [F_ST_/(1 – F_ST_)] among the twelve populations of wild and domestic *C. medica*. F_ST_ was calculated according to Weir and Cockerham (1984).
